# Photo-elicitation with adolescents in qualitative research: an example of its use in exploring family interactions in adolescent psychiatry

**DOI:** 10.1186/s13034-017-0186-z

**Published:** 2017-10-06

**Authors:** J. Sibeoni, E. Costa-Drolon, L. Poulmarc’h, S. Colin, M. Valentin, J. Pradère, A. Revah-Levy

**Affiliations:** 1 Service Universitaire de Psychiatrie de l’Adolescent, Argenteuil Hospital Centre, Argenteuil, France; 2 ECSTRA Team, UMR-1153, Inserm, Paris Diderot University, Sorbonne Paris Cite, Paris, France

**Keywords:** Photo-elicitation, Adolescence, Family functioning, Qualitative methods, Methodology

## Abstract

**Background:**

Photo-elicitation is a method used increasingly often in qualitative health research, and its positive effect on the research process is well established today. Photo-elicitation appears to facilitate verbalization and insight and to improve relationships between the researcher and participants, thereby enriching the quality of the data collected. Nonetheless, it is barely used at all in the field of adolescent psychiatry. With the aim of exploring the potential of these methods for research with adolescents receiving psychiatric care, we conducted a qualitative photo-elicitation data collection study with this population, asking them about family interactions around food.

**Methods:**

The data were collected from 15 adolescents and 17 parents during semi-structured interviews in which a photo taken by the adolescent served as the focus of discussion. Data were explored through inductive thematic analysis.

**Results:**

Photo-elicitation played a threefold role in this study: (1) it induced the teens’ interest, thought, and pleasure, (2) it played a mediating function during the interviews, and (3) it enabled family interactions to be viewed from the adolescent’s perspective. Three themes concerning family interactions were found: (1) parent–child relationship patterns, (2) the functioning of the family group, and (3) the adolescent’s individual relation with food, that is, the issue of the adolescent’s autonomy.

**Conclusions:**

Photo-elicitation proved to be an innovative technique in qualitative research in the area of adolescent psychiatry, one that enriched the data and enabled the emergence of new themes in this field, related in particular to the process by which adolescents develop autonomy.

## Background

Visual narrative research methods are used increasingly often in the field of qualitative health research. Derived from work in visual anthropology, photo-elicitation involves the use of photographs as support during a research interview [1]. Currently, the participants themselves most often take these pictures. The positive effects of photo-elicitation on the research process have been widely described in qualitative literature studying adults. It appears to improve the quality of the data collected [2] by promoting active cognitive involvement and better participation in the research [3]. The principle of photo-elicitation *empowers* participants, by putting them in a more active position and thereby giving them the opportunity to influence the research process more strongly [4]. Photo-elicitation may also facilitate the construction of a bond between participants and researchers [5] and may promote verbalization of thoughts and emotions [6].

In recent years, qualitative health research has also been developing among adolescents. The qualitative approach makes it possible to consider adolescents as active participants in research, to recognize their right and autonomy of thought and to give them a voice [7]. Qualitative research within this population nonetheless raises specific questions, in terms of both ethics [8] and methodology [9], including the use of visual methods [10]. Qualitative research with adolescents in the general population requires consideration of the developmental aspects of this life stage, both cognitive and affective, and of the anxiety inherent in the situation, the imbalance in the researcher–adolescent relationship, the adolescents’ lack of involvement for the research, and the adolescents’ difficulties—common at this age—in expressing themselves and especially their emotions verbally [9]. These points are even more salient when the adolescent presents a psychiatric disorder. The methodological literature describes adolescents with psychiatric disorders to be “doubly vulnerable persons” [11], with “multi-faceted vulnerability” [12], and qualitative research in this population is considered a methodological challenge. Moreover, certain psychiatric symptoms, either cognitive or affective, may directly affect the interview. It is therefore difficult to obtain a detailed and deep narrative of experience from this group population. Accordingly, many qualitative studies exploring psychiatric issues of adolescents involve interviews with parents, caregivers, or physicians. At the same time, mental health professionals in general and those working with adolescents in the mental health field in particular endeavor to take into account the needs of the patients and take their subjective health status into consideration [13].

The literature already includes several qualitative studies of adolescents that used photo-elicitation [14, 15]. Nonetheless, photo-elicitation has not been used in research in the field of adolescent mental health, with the exception of a qualitative study of the school experience of adolescents with autistic spectrum disorders [16]. In an earlier study, we used photo-elicitation to explore the role of food in the family relationships of obese adolescents [17]. The question of food and the family meal appeared relevant for exploring these adolescents’ family interactions, consistent with the data in numerous studies that have demonstrated the important role of food in family interactions among adolescents. On the one hand, research has shown that factors such as parental dietary preferences, family meal structure, and single parenthood can influence body mass index (BMI) in childhood and especially adolescence [18]. On the other hand, many authors have described the important part food plays in family interactions, as seen both in the consideration of the act of nurturing [19] and in the issues of power and control that arise between parents and adolescents around food [20].

We have focused for several years on the crossed perspectives of care in adolescent medicine and psychiatry—the views of teens, their parents, and the professionals providing them with care [17, 21, 22]. In our study using photo-elicitation with obese adolescents and their parents, we obtained an elaborate narrative about food and family interactions from both the groups. In line with this study, we used the same design among adolescents receiving psychiatric care to examine whether this visual method of photo-elicitation is an effective tool for exploring family interactions with adolescents receiving care for psychiatric disorders unassociated with food and with their parents. Furthermore, exploring family interactions among adolescents receiving psychiatric care is an important issue in the practice of adolescent psychiatry. Regardless of the disorders presented, this exploration most often provides new insights that illuminate both evaluation and treatment perspectives.

## Methods

Table 1 presents the overall study design in detail. This exploratory multicenter study used a qualitative methodology: sampling was purposive [23]; Adolescents were asked to take a photograph of a family meal that would subsequently be discussed in two individual interviews a week later, first with the adolescent, and then separately with one or both parents; data saturation was achieved according to the principle of theoretical sufficiency [24]; and a five stage thematic analysis was used to explore the data [25] (Table 2). This study complied with the COREQ guidelines [26].

**Table 1:** Study design

Qualitative approach	Phenomenology
Research paradigm	Constructivism
Setting	Study developed in a research group seeking to develop the use of qualitative research in adolescent psychiatry
Ethical issues	The relevant French Institutional Committee of the Paris North University Hospital Group approved this study All patients and their parents provided written *consent* before inclusion
Sampling strategy	Purposive sampling strategy: selective and deliberate
	Researchers first contacted clinicians at recruitment sites (Argenteuil and Remiremont Hospitals) where recruitment was planned and explained the study design and objectives to them in detail
	Clinicians identified potential participants—adolescents and parents—whom they considered most likely to provide useful information
	Clinicians mentioned the study to potential participants and gave them an information sheet about it
	Researchers met each interested teen and his/her parents
	To describe the study
	To collect social and demographic data
	To obtain their written consent
Inclusion/exclusion criteria	Adolescents between 12 and 18 years at the time of the interview
	Adolescents and parents must speak French fluently
	Adolescents must not have an eating disorder (i.e., anorexia nervosa, bulimia, avoidant/restrictive food intake disorder, or another unspecified eating disorder) or a weight-related disorder such as obesity
	Adolescents could have food-related symptoms and their effects on the family relationships would be part of our field of exploration
	Adolescents must not present acute or severe psychiatric disorders—schizophrenia, bipolar disorder, or autistic spectrum disorders—(the focus of this study was not the adolescents’ psychopathology but rather the relevance of photo-elicitation in research in adolescent psychiatry)
	Families must not have major dysfunctional patterns, such as neglect or abuse
	Adolescents must be able to talk about their experience of family relationships around food and the family meal
	Adolescents must have been receiving care for at least 6 months
Participants	Adolescents receiving psychiatric care in an outpatient setting and one or both of their parents
	All saw their psychiatrist at least once a month
	All had chronic mental disorders that had begun during adolescence (depression, anxiety, social phobia, personality disorder). This diagnosis was made by each patient’s referring psychiatrist, according to DSM 5 criteria
	None had a somatic disease
Data saturation	Data saturation according to the principle of theoretical sufficiency:
	When new participants were not adding anything significant to the database
	When the themes obtained offered a sufficient explanatory framework in view of the data collected
	Two further individual interviews were conducted with no new themes emerging, to ensure full data saturation
Data collection period	From April 2015 to November 2015
Data collection methods	Individual in-depth interviews using photo-elicitation:
	At the end of the preliminary interview, the adolescent was given a digital camera. They could refuse and use their own equipment (smartphone) if they preferred
	Instructions: *“You must take a photograph of the table after a family meal. The table should not yet have been cleared. No person should appear in the picture, so everyone at the table must have gotten up. You can take as many pictures as you want, but you will have to choose just one that you will talk about with the researcher at the interview”*
	We chose to ask for a photo after the family meal to encourage a narrative of the entire meal
	For ethical reasons, no person could appear in the photographs
	Individual interviews a week after:
	Of the adolescent and immediately after of the parent(s)
	The selected photograph was displayed on a computer screen during both interviews
	The interviewer began by asking the adolescent for a description of the family meal from which the photograph resulted
	At any point during the interview, the interviewer and the participant could go back to the photograph
	Individual in-depth interviews:
	Unstructured, open-ended approach
	One introductory prompt: “can you tell us about this family meal?”
	To get rich and detailed personal data from each participant
	To enter the interviewees’ psychological and social world
	To remain open and attentive to any unknown issues that they might introduce
	All interviews were:
	Audio-recorded with participants’ permission
	Transcribed word for word, including nonverbal aspects (pauses, laughter, etc.)
	Anonymized
Interviewers	The same researcher (JS), an adolescent psychiatrist, conducted all the interviews
Duration of the interviews	From 60 to 90 min
Data analysis	Thematic analysis:
	To identify, analyze and report themes within data
	To identify the similarities and the differences in the participants’ narratives
	To discern recurrent patterns and to integrate new elements that emerged from the analysis
	In a data-driven analysis with inductive approach = coding the data without any reference to theoretical notions or researcher’s preconceptions
Criteria to ensure validity	Analysis conducted independently by the three researchers (JS, EC, LP)
	To verify that the themes identified were an exact reflection of the data
	Research group monthly meetings:
	To discuss the results
	To be supervised by a researcher more distant from the material (ARL)
	To resolve disagreements on the inclusion or exclusion of a theme (discussion continued until a consensus was reached)

**Table 2:** Process of inductive thematic analysis

	Activities	Rationale
Stage 1	Repeatedly read each transcript, as a whole	Obtain a global picture of the interview and become familiar with the interviewee’s verbal style
Stage 2	Code the transcript by making notes corresponding to the fundamental units of meanings	Make descriptive notes using the participant’s own words
Stage 3	Make conceptual notes through processes of condensation, abstraction, and comparison of the initial notes	Categorize initial notes and reach a higher level of abstraction
Stage 4	Identify initial themes Provide text quotes that illustrate the main ideas of each theme	Themes are labels that summarize the essence of a number of related conceptual notes
Stage 5	Identify recurrent themes across transcripts and produce a coherent ordered table of the themes, gathered into domains of experience	Move from the particular to the shared across multiple experiences. Recurrent themes reflect a shared understanding of the phenomena among all participants

The study included 15 adolescents, 10 girls (F) and 5 boys (M). Table 3 summarizes their characteristics. A total of 17 parents were also interviewed giving data from a total of 32 participants. All the adolescents recruited agreed to participate. Nonetheless, some parents refused to be interviewed; some explained that food was a subject too personal and private to be shared or, on the contrary, that the subject was neither interesting nor relevant. Numerous fathers shared the latter opinion and chose not to participate. In the families in which the parents were separated, the parents not having primary custody did not want to or could not participate.

**Table 3:** Adolescents’ characteristics

	Gender	Age	Body mass index (kg/m^2^)	Psychiatric diagnosis	Adjunctive treatment	Duration of treatment in months	Parental situation	Parents interviewed
F1	Girl	18	19,6	Depression	Sertraline 100 mg/day	22	Divorced	Mother
F2	Girl	17	23	Anxiety disorder	Individual psychotherapy	8	Divorced	Mother
F3	Girl	16	21	Borderline personality disorder	Individual psychotherapy, day hospital	18	Divorced	Mother
F4	Girl	14	18.4	Anxiety disorder	Fluoxetine 20 mg/day	8	Married	Father
F5	Girl	13	21	General anxiety disorder	Individual psychotherapy	14	Married	Parents
F6	Girl	16	22.3	Panic disorder	Individual psychotherapy	12	Divorced	Mother
F7	Girl	17	21.5	Borderline personality disorder	Individual psychotherapy	6	Married	Parents
F8	Girl	15	19.6	Depression	Individual psychotherapy, fluoxetine 20 mg/day	11	Married	Father
F9	Girl	16	18.3	Depression	Individual psychotherapy	16	Divorced	Mother
F10	Girl	14	20.6	Panic disorder	Sertraline 100 mg/day	7	Divorced	Mother
M1	Boy	13	22.7	Depression	Fluoxetine 20 mg/day, day hospital	24	Divorced	Mother
M2	Boy	16	19.1	Borderline personality disorder	Individual psychotherapy	15	Divorced	Mother
M3	Boy	17	18.7	Depression	Fluoxetine 40 mg/day, day hospital	24	Divorced	Mother
M4	Boy	15	21	Depression	Individual psychotherapy	16	Married	Father
M5	Boy	16	19.6	Borderline personality disorder	Individual psychotherapy, day hospital	15	Divorced	Mother

## Results

We present first the results of the role of photo-elicitation in the research process and then the results about the family interactions around food. Extracts of the transcripts have been selected to exemplify the themes described and transcribed in English for the sole purpose of this article. All personal information has been removed, to protect the confidentiality of the participants.

### The role of photo-elicitation

We observed that using photo-elicitation added specific, original input to this project with adolescents on the issue of family. First of all, they were invested in the task given to them and showed creativity, thought, and also feelings of pleasure in taking the photograph. Next, this picture did indeed serve a mediating function during the interview, both for the participants and the interviewer. Finally the task assigned to the adolescent, to take this picture, was itself the object of family interactions.

#### Take a picture: participate in this study with pleasure and engagement

The teens’ act in taking the picture by itself facilitated their commitment to the study. Better, they were all invested in the task assigned to them. All reported thinking about the production of the photograph. Some mentioned a desire to show the most or the best, others described esthetic concerns about the questions of light, color, or symmetry of the objects on the table, and especially about the shot selected.

F1: “I chose that one because it’s beautiful, because I did it really well.”

Taking the photo and then choosing it required a thoughtful effort that included anticipating and imagining the conversations of the research interview.

*M4: “Finally I opted for seeing everything that I usually see, so that we can do the best examination of the picture.”*

The adolescents sought with their pictures to reflect their experience as closely as possible. They were thus able to impose their point of view on the scene and directly influence the research process.

*F8: “I chose this picture because it is exactly what I see from my seat, it’s taken exactly as if it were my eyes.”*

Beyond their investment in the study, the teens also enjoyed performing this task. Taking only one photograph did not suffice to express the enthusiasm for this project.

*M2: “Why were we limited to a single picture? Me, I took lots of them, with a zoom, from above (…) do you want to see them?”*

Parents also explicitly mentioned the child’s pleasure and investment.

*Mother of F1: “We were taken aback the day that she said, ‘don’t clear the table, I have to take a picture.’ I don’t know if her stepfather was more astonished by the story of the photo or by her attitude, how she took it to heart.”*

#### The photographic image: a support for the narrative

During the interviews with the adolescents, the photo was at the center of the verbal exchanges. The presence of the photo as the basis for the conversation made it possible to disinhibit the adolescent-researcher relationship.

*F5: “I would never have imagined you could say so much about a photo!”*

The teens leaned on the photo to verbalize their memories and their emotional experiences.

*M5: “Do you see this fruit basket at the center? eh, I made it in school, for Mother’s Day, when I was in kindergarten.”*

The researcher also used the picture to facilitate conversation and to approach a new subject.

*Interviewer: Whose plate is that, with the no*-*fat yogurt?*

*F2: “Ah that’s my mother’s, they are her yogurts and no one else can touch them!”*

Finally, the photograph embodied the teen’s point of view during the interviews with the parents.

**Fig. 1:**
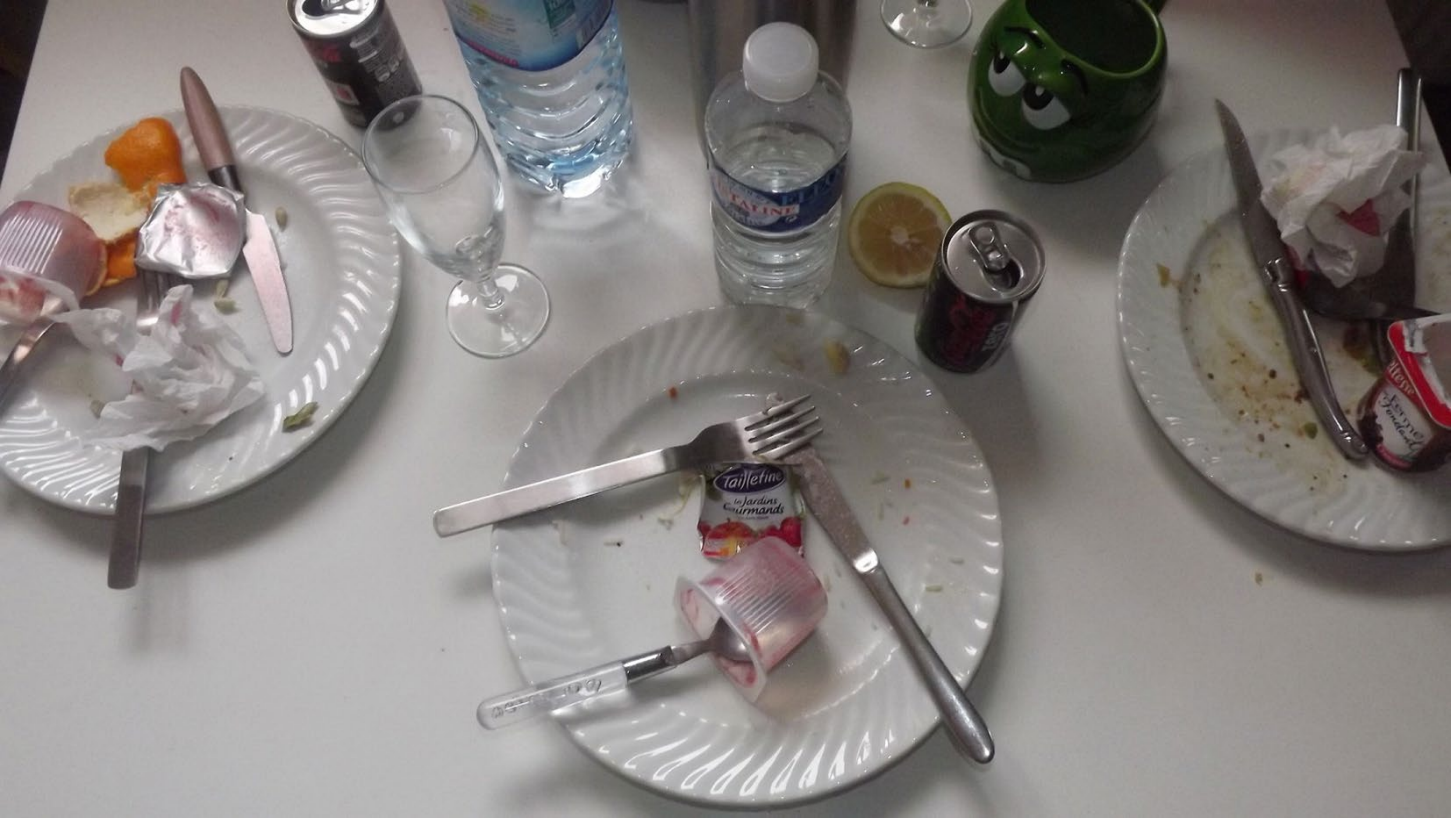
F3’s photograph

*Mother of F3 (about Fig.* *1* *): “I was wondering why she chose to keep that one; she took others, better … at least, in my opinion.”*

#### Access to family interactions through the object that was photographed

##### *The taking of the picture*

The task assigned to the teen often led to conversations in the families and became a family task. That is, the entire family felt concerned and gave advice, either at the teen’s request, or spontaneously.

*M2: “I think my little brother also wanted to take pictures; so I asked for his advice and he was so happy. He had the idea of taking pictures of our two plates to show the difference, but my mother said it would be better to be able to see the whole table.”*

Sometimes parents had exercised a right of oversight or censorship, illustrating the issues of control and asymmetry in the adolescent-parent relationship.

*M1: “Isn’t my dog’s head in this picture? Ah no! But at the beginning I had kept it but my mother must have deleted it when she was checking.”*

Taking the picture also gave some adolescents the opportunity to assert themselves within the family as the person to whom this task was assigned.

*F4: “…it was me! They were there, but I am the one who chose and who took the pictures.”*

##### *View of the family visible in the still*-*life photograph*

The image most often let us see a view of the family and of the family functioning—a view proposed by the adolescents, either by the choice of a specific meal to photograph or by the specific shot. Some teens chose to photograph the only meal where the entire family was together, thus noting the rarity of these moments and the lack of family communication and cohesion.

*F1: “A meal where we are all together because on Monday my mother has English and I have dance in the evening (…) Tuesday we are all together.”*

**Fig. 2:**
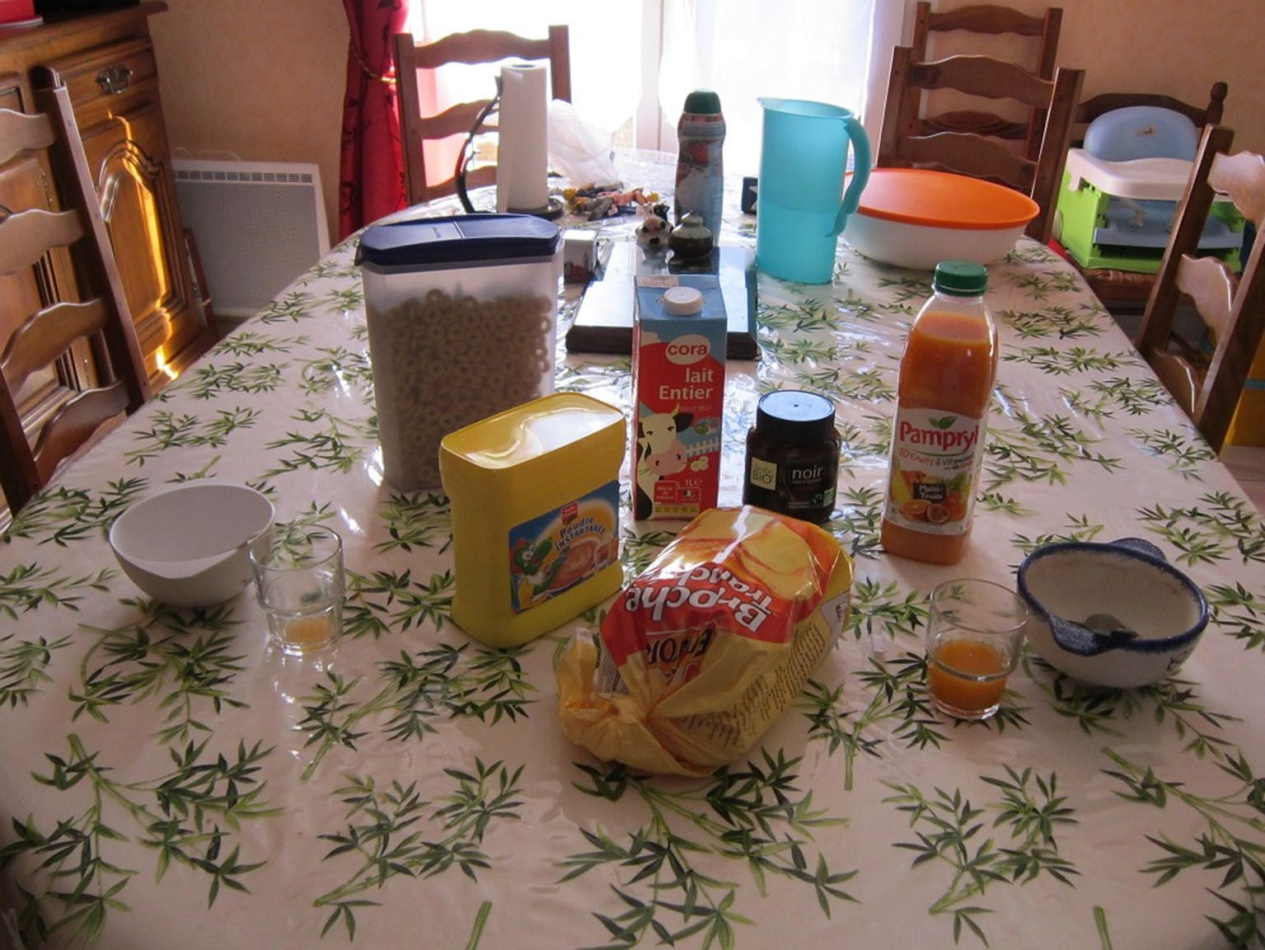
F4’s photograph

Other adolescents on the contrary stressed a particular relationship in adapting the instructions and choosing a meal where only some family members were present .

*F4 (Fig.* 2 *): “ Breakfast with brother and sister… it’s an important moment for us, when the two of us are really together.”*

Finally, some adolescents choose a particular shot to illustrate the family history. The view of the photograph taken by F3 (Fig. 1) gives the impression of a horizontal slice. She presents a view from above of a table with three plates, but shot such that the viewer cannot tell how the table continues and whether or not there is a fourth plate outside the field of view. She used this project to illustrate her distress about the separation of her parents, a recurrent topic during the interview.

*F3: “I don’t know why I kept this picture. It’s true that the framing is bad, you could say it’s cut (…) But this table doesn’t hold four people. When my father still lived with us, we didn’t eat there anyway, we ate in the living room.”*

### *Exploration of family interactions around meals and food*

The analysis of the interviews allowed us to identify three themes concerning family interactions around food. The first concerns parent–child relationship patterns, the second the functioning of the family group, and the third the adolescent’s individual relation to food and therefore the process of separation from the family.

#### Parent–child relationship patterns

##### *Express both difference from and resemblance to others by food*

We first found in these adolescents a desire to differentiate themselves from their parents through what they eat, but also to confirm that they belonged to their family and claimed its heritage. They signaled this continuity explicitly by appropriating the parental discourse about food.

*M4: “In fact, it works like that, I have to taste everything each time. My mother repeated it incessantly and now it’s like her voice is in my head.”*

The differentiation could be observed through the adolescent’s new tastes, most often accompanied by attraction to a cuisine different from that of the family. This issue of difference and resemblance was clearly illustrated by the adolescents’ acts of cooking. Some reproduced family recipes, other compromised with a variant of a basic family dish, while others invented completely different recipes to demonstrate their individual relation to food.

*M1: “I began to invent recipes, just for me. Once I made a mixture of pears and potatoes in the blender.”*

##### *Food: expressing love within the parent*–*child relationship*

Food was a way of expressing love within the family. Mothers made this discourse explicit.

*Mother of F9: “there’s love in it, it’s nothing but love (…) because they know I sacrificed to make it…”*

Adolescents also considered food as a way of expressing love.

*F5: “I don’t really like boiled beef (…) I make myself eat a little, because I know it makes her happy when I do.”*

#### Functioning of the family group

##### Family cohesion and a relational game

The family group experienced authentic cohesion around the family meal. These moments were special because they were together and sharing. Food was actually secondary and was sometimes only a pretext for getting together.

*F10: “Sometimes the meal is over but we stay there, we sit, and we talk.”*

Family cohesion during meals was especially the foundation of a relational game within the family, a flexible game that allowed the expression and sharing of all sorts of emotions.

*Father of F7: “That can be two minutes of screaming and two minutes later we are all going to laugh.”*

Everything was part of the game. The teens refused to be at the table with their parents and played at not eating but nonetheless ended up eating. The parents were perfectly aware of this.

*Mother of F6*: *“There was a phase when she didn’t eat, at least, not in front of me, but there was this strange phenomenon of food that disappeared from the refrigerator.”*

The teens also played with parental control around food. They could break the rules, but it was still part of the game.

*M2: “In fact, I have packages of chips hidden in my room (…) my mother yells at me but at the same time she laughs because she did the same thing when she was my age.”*

##### *Transmission and family history*

Food also served a function in the transmission of family history.

*Father of F4: “We are epicureans, when we get together for these occasions, and we have to transmit that to the children.”*

This implied first of all transmission of the family history, based on ways of cooking things, special recipes that were transmitted from generation to generation and carried with them the culture of the family.

*Mother of F5: “There was also Grandma Alice’s apple charlotte, there’s something special when I cook these recipes.”*

Food also gave access to the current family situation: reorganizations of family life were illustrated by changes in food or diet.

*M1: “My father never let us have onions or butter. Now for example, we can make onion tarts often.”*

For the adolescents, parental separation furnished two parallel histories, and food could serve as a witness to both and thus confirm the separation.

*F9: “My father is kind of random, my mother very straightforward. My mother is steak, salad, yogurt, and an apple; my father is an omelet and chips and then merguez mixed with anything.”*

#### An individual relation to food

This theme was unexpected in our exploration of family interactions around food, but we found that adolescents asserted their own individual relations to food. It did not involve simply claiming their own tastes in food but also deciding when, where, and with whom to have meals. The adolescents showed that they wanted to choose and make decisions about food based on their own experience.

*F7: “I think that my parents never made me eat something, so I always said, I don’t like it. But now I try, I verify if I really don’t like it….. and sometimes I like it and sometimes I don’t.*

They showed a desire to cook for themselves even if they remained attached to the family cuisine.

*Mother of F6: “In fact, she is totally ‘I can make it but I’d prefer if you do it.”*

In fact, the transmission of culinary practices was a progressive movement toward autonomy, preparing the teen for a future life outside the family home. These practices were thus transmitted in several ways, from a passive *watch it being done* to *I’m making it all by myself*.

*F4: “First, I watched my mum and then she showed me and after that she let me do it, but stayed behind me. After that, she just watched and now I do it alone.”*

The individual relation with food was also found in the adolescents’ interactions with their peer groups.

*F4: “With my friends, we talk a lot about food, we organize ‘crepe parties’ just for us, and it’s great.”*

Finally, the parents too asserted their own individual relation with food. They had their own tastes and desires in food and refused to sacrifice them.

*Mother of F3: “Yes, I cook brussels sprouts, I cook them for me; it would bother me to think that I deprive myself of something I like because of my daughters’ tastes… after all, the fridge is big enough.”*

## Discussion

The main objective of this study was to examine the value and feasibility of using photo-elicitation in research in adolescent psychiatry via an exploration of the role of food in family relationships.

Photo-elicitation appeared to be feasible in adolescent psychiatry research and helpful for interviewing teens with diverse psychiatric disorders. There are three aspects especially important to point out here.

First, taking the photo promoted the adolescents’ involvement in the project and generated a positive feeling toward it. Some authors suggest that modern vocabulary and contemporary modes of expression are useful in research interviews with adolescents [8]. The use of the photo-elicitation tool fits into this approach. Photography is a favored mode of expression for youth, occupying an important place in their daily lives, in particular in their social networks. Here, 11 adolescents spontaneously refused to use the cameras we planned to give them and preferred to use their own smartphones. This idea also appears in the study by Yi-Frazier et al. [15], they asked teens with diabetes to use Instagram—a social network whose primary medium is photographs—as a form of photo-elicitation for their study.

Second, most adolescents are quite skilled at photography, and this gives them the opportunity to better express their point of view [9]. Accordingly, as Mack et al. [8] wrote, “*Research will be a positive experience for adolescents when they know that their input is important and valued”.* We consider that in this study, the teens were fully able to influence the research process because, although we initially sought to focus on the family interactions around food, our most original result concerns the adolescents’ individual relations with food. Our study fits within the constructivist paradigm, and the visual method helped to co-construct the results [27]. Our methodological choice to use photo-elicitation—and probably also our instructions—empowered the adolescents, by asking them to perform an action they were skilled at and comfortable with, to reveal their own vision. We placed them in the position of author. This position enabled the emergence of a theme focused on the issue of the adolescent’s empowerment in the construction of his or her own self.

A last point about the photograph is that the teens experienced and expressed pleasure in taking the picture, choosing it, showing it, and talking about it. Sutton et al. [28] argued that the presence of pleasure increases the success of study recruitment. We note that all the adolescents who were asked to participate in this research project agreed to do so. In her review of the literature about qualitative research with children and adolescents, Kirk [9] concluded that it is important to use child-friendly techniques so that the participants can have fun during the data collection.

Tested in our study, photo-elicitation was a tool that enabled us to obtain rich narratives of experiences that led to innovative results. Two of the themes in our results (parent–child relationship patterns and the functioning of the family group) have also been found in studies of obese adolescents [17, 29]. The literature describes the cohesive function of the family meal [18], like that of food, as a vector of transmission of the family history and culture [30]. These dimensions are above all cultural and are related to family structure in Western countries. The third theme, which shows adolescents’ individual relation to food, is an original result of our study. That is, the adolescents insisted on their taste in food and their own attitudes towards it. They consider themselves the authors of their food-related actions and choices. This result can be linked to the issue of identity construction in adolescence, especially through the idea of *self-concept* [31]; this notion underlines the importance of the definition individuals give to themselves, how they perceive themselves. This idea of *self*-*concept* in adolescence has been developed in the recent literature, both in a cognitive, neurobiological dimension [32] and in an environmental perspective [33]. The adolescent’s *self*-*concept* is constructed from multiple dynamics: his or her individual society, peer group, and family [34]. Food may be an accessible marker of this potential identity construction. These links between identity construction and food have already been described in sociology in relation to the general population of adolescents [35, 36]. From a methodological perspective, this result also shows that photo-elicitation can be used to identify and explore dynamic examples of self-concept and identity construction.

To the best of our knowledge, no study in the field of adolescent psychiatry has described the importance of this individual relation with food in a population of adolescents with a variety of psychiatric disorders. This particular context raises a question: is this preoccupation of adolescents about their food-related desires and choices linked to the adolescent process of identity construction, or is it a marker of treatment that may have promoted the adolescent’s autonomy? The development of the adolescent’s *self*-*concept* may be considered, in the latter case, as a treatment effect, resulting from the various kinds of care he or she has received.

### Implications for adolescent mental health research

As we mentioned above, qualitative research among adolescents with psychiatric disorders is considered as a methodological challenge. It is already clear from the literature that photo-elicitation is a methodologically relevant choice with adolescents, with many advantages: greater control over the visual and verbal discourse, easier relationship between researcher and adolescent, greater influence on the research process [10, 37, 38]. Yet, its potential interest with adolescents with psychiatric disorders has not previously been explored. Our results highlight the positive aspects of using this tool with this specific population as well as its methodological relevance in qualitative research among them.

### Limitations

One limitation is inherent to photo-elicitation as a tool and its generalizability. Its use may be restricted to teens able to take pictures, although we found no teens in our sample who were unable to do so and it is limited to teens with reasonably good vision.

Two other limitations concern the results of the analysis of the content of the patients’ experience. The first is the difficulty in determining whether results are specific to our population of adolescents receiving psychiatric care or if, instead, they might be true for all adolescents. Certainly, the advantages of using photo-elicitation with this age group and its use as a vehicle for discussing family functioning seem clear and are not specific to our study population. To verify these assertions, however, would require an identical qualitative study in the general population of adolescents, and then a quantitative research design with matched comparison groups from the general population. The second limitation involves the diagnostic heterogeneity of the members of our sample: 6 with depression (3F:3M), 4 with borderline personality disorder (2F:2M), 2 with anxiety disorder (2F:0M), 2 with panic disorder (2F:0M), and 1 with generalized anxiety (1F:0M). We thus cannot prejudge the relevance of our results in particular clinical situations.

## Conclusions

This qualitative study used the tool of photo-elicitation to explore the family interactions around food in adolescents receiving psychiatric care. From the methodological perspective, our results simultaneously illustrate the value of developing qualitative research in adolescent psychiatry and the need to adapt this research to this specific population by using innovative and original techniques that enable teens with psychiatric disorders to express their subjective experience.

## Abbreviation

BMI: body mass index
